# Supplementary figures and images for: RNF8 Dysregulation and Down-regulation During HTLV-1 Infection Promote Genomic Instability in Adult T-Cell Leukemia
Source: PLoS Pathog. 2020 May 26;16(5):e1008618. doi: 10.1371/journal.ppat.1008618 (PMC7274470; doi:10.1371/journal.ppat.1008618)

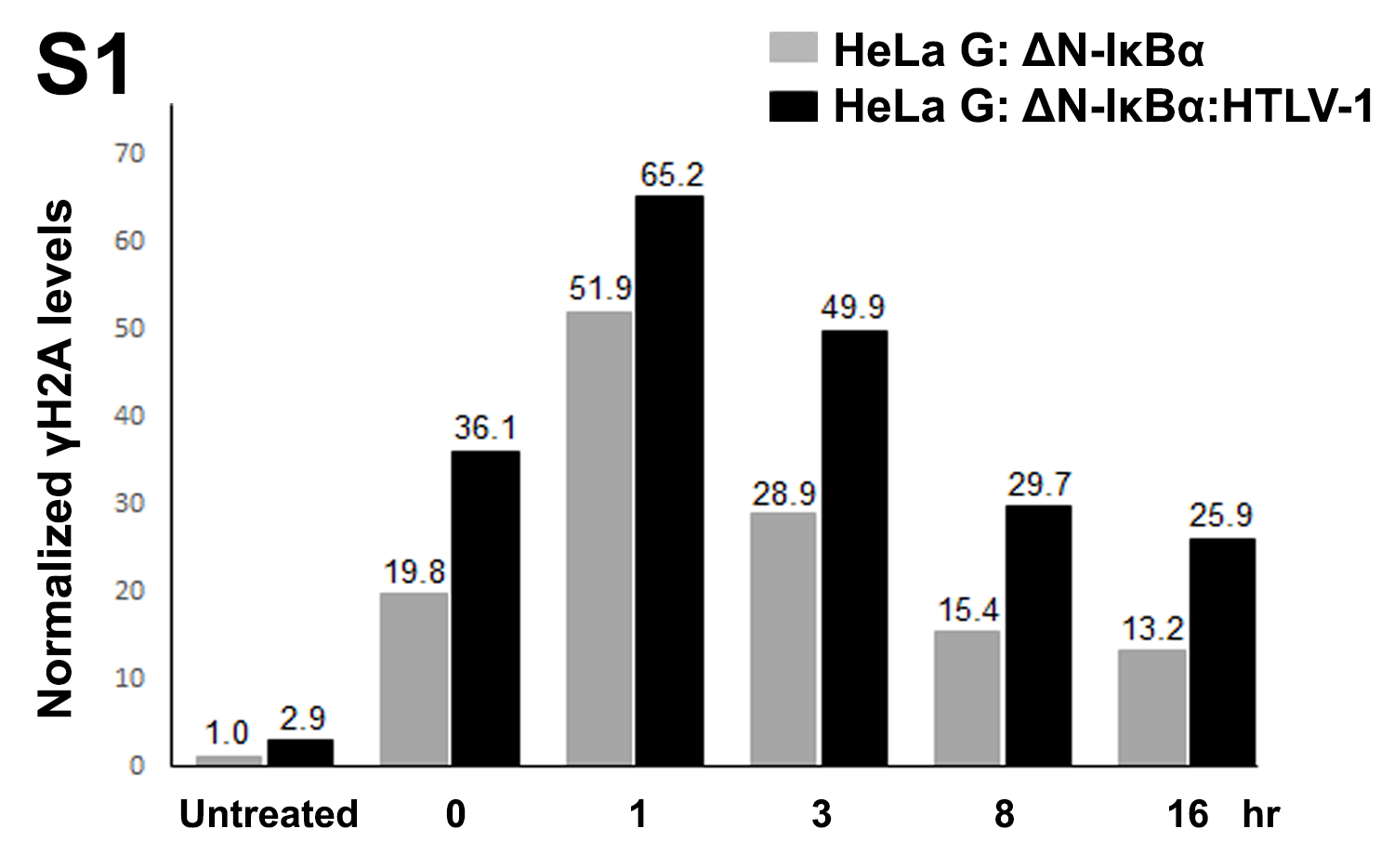

Supplement: S1 Fig — γH2AX levels of HeLa-G: ΔN-IκBα and its progeny HeLa-G: ΔN-IκBα:HTLV-1 cells in Fig 1A were quantified using Image J and normalized to the β-actin (Actin) loading control. The values were presented relative to the untreated HeLa-G: ΔN-IκBα. (TIF) [file ppat.1008618.s001.tif]

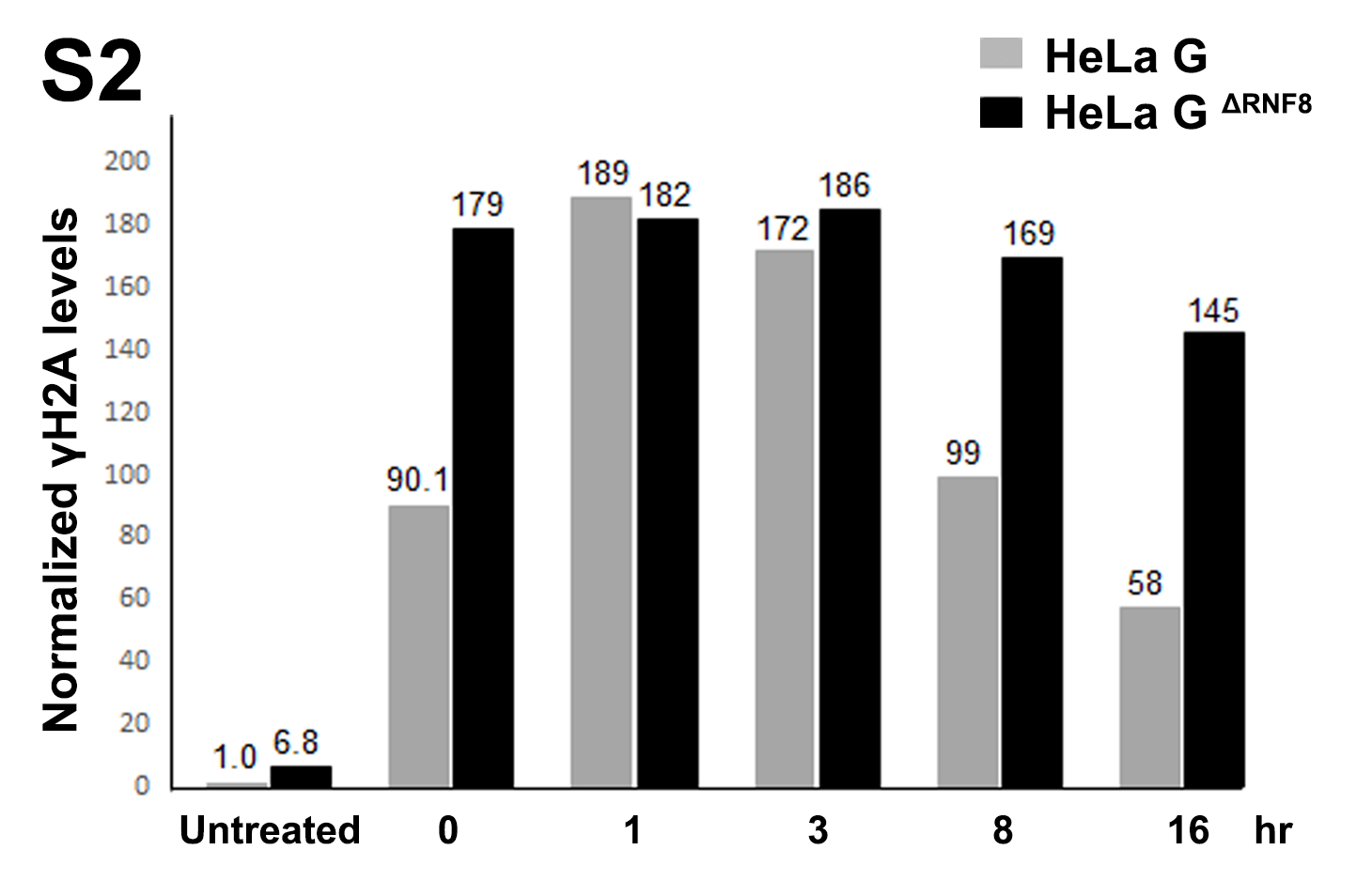

Supplement: S2 Fig — γH2AX levels of HeLa-G and HeLa-GΔRNF8 cells in Fig 1B were quantified and normalized in S1. The values were presented relative to the untreated wild-type HeLa-G control. (TIF) [file ppat.1008618.s002.tif]

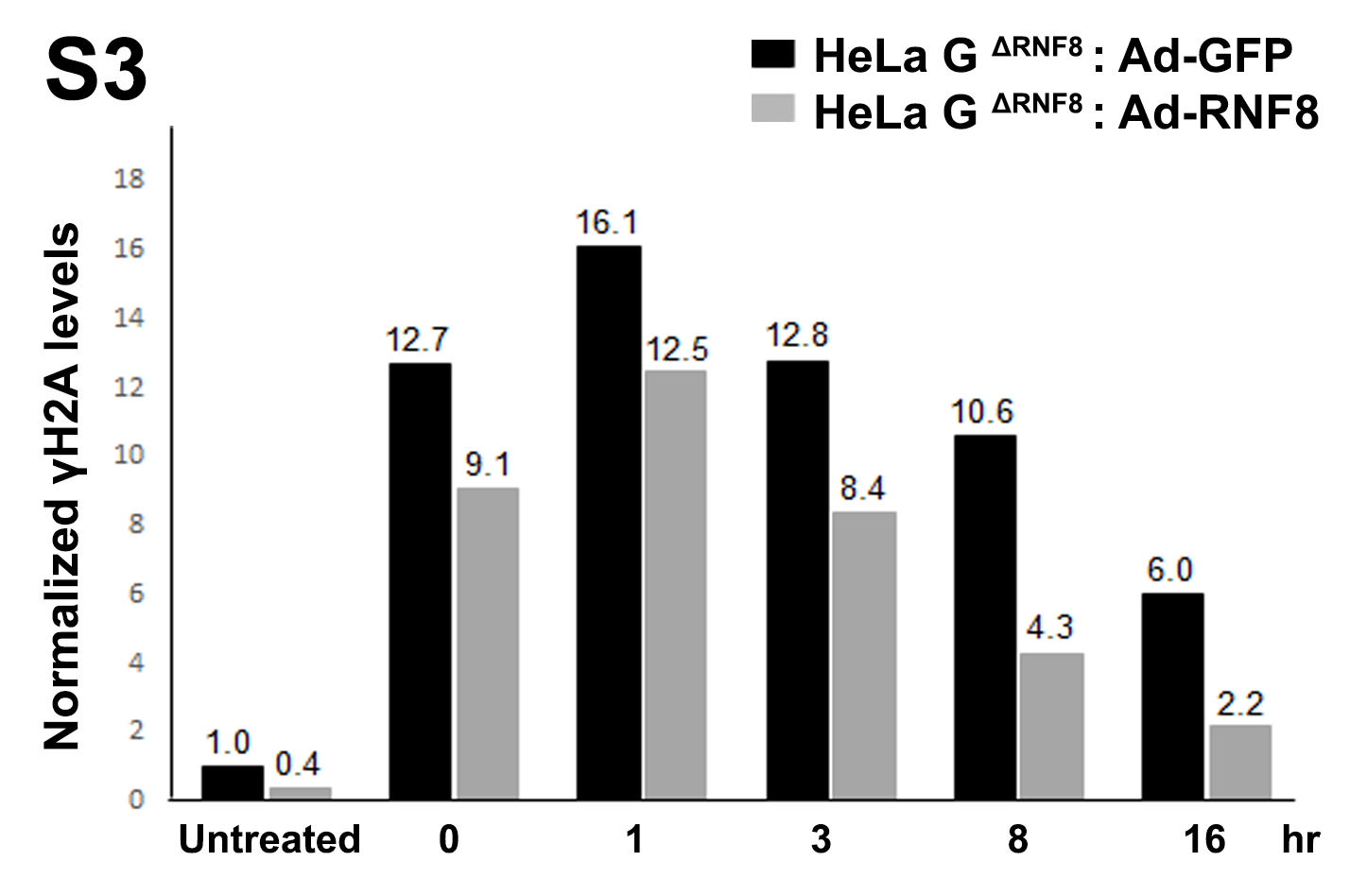

Supplement: S3 Fig — γH2AX levels of HeLa-GΔRNF8 cells transduced with either Ad-GFP (Control) or Ad-RNF8 in Fig 1C were quantified and normalized as above and presented relative to the untreated HeLa-GΔRNF8 cells transduced with Ad-GFP. (TIF) [file ppat.1008618.s003.tif]

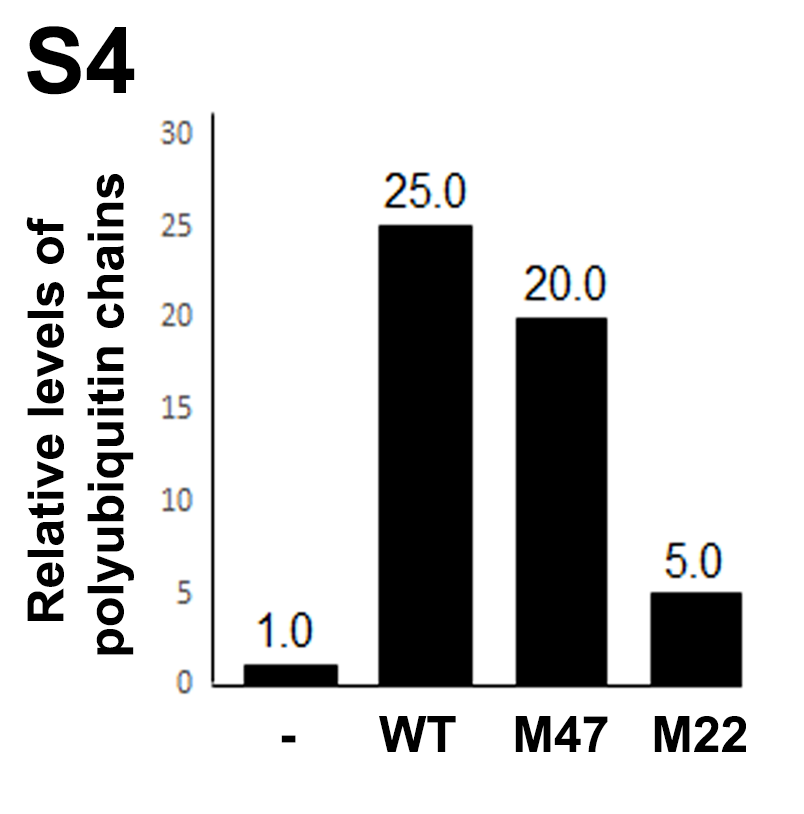

Supplement: S4 Fig — The total polyubiquitin chain signals in Fig 3A were quantified using Image J and normalized to the β-actin (Actin) loading control of each experiment, and then to the normalized value of the untransfected control cells (denoted as “-“). (TIF) [file ppat.1008618.s004.tif]

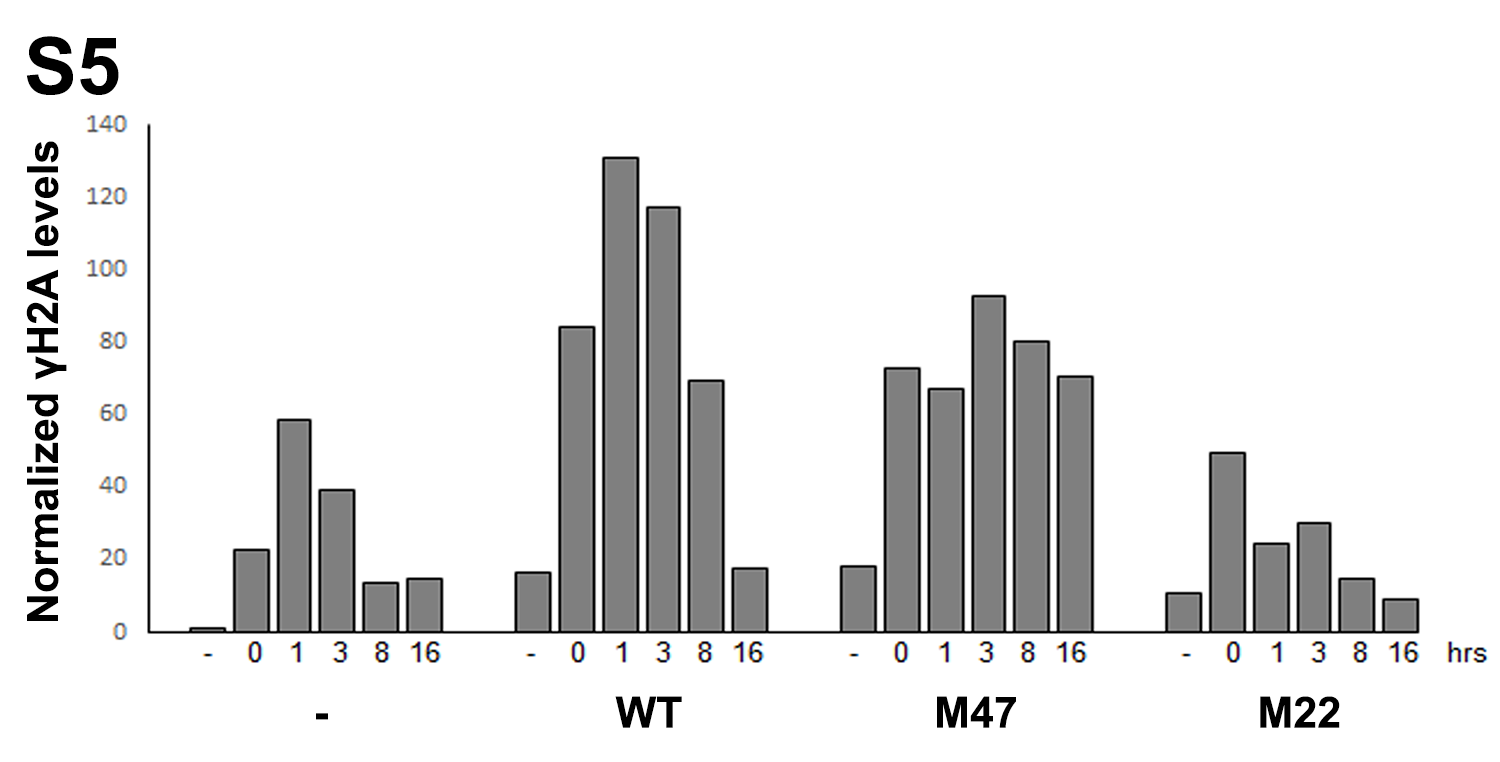

Supplement: S5 Fig — γH2AX levels of HeLa-G: ΔN-IκBα and its progenies expressing WT, M47 and M22 Tax were quantified using Image J, normalized to the GAPDH loading control and then to the untreated sample of HeLa-G: ΔN-IκBα cells. (TIF) [file ppat.1008618.s005.tif]
