# Supplementary material for: RNF8 Dysregulation and Down-regulation During HTLV-1 Infection Promote Genomic Instability in Adult T-Cell Leukemia
Source: PLoS Pathog. 2020 May 26;16(5):e1008618. doi: 10.1371/journal.ppat.1008618 (PMC7274470; doi:10.1371/journal.ppat.1008618)
Supplement: Attachment — Submitted filename: Cover letter-Response to Reviewer Comments-final-043020.docx [file ppat.1008618.s006.docx]

April 30, 2020

Dear Editor:

#### We hereby submit the revision of a manuscript entitled “RNF8 Dysregulation and Down-regulation During HTLV-1 Infection Promote Genomic Instability in Adult T-Cell Leukemia” for further consideration. We thank the reviewers for their cogent comments, which have helped us complete the study. We would also like to express our sincere appreciations for the tireless efforts of the reviewers and the editorial staff of PLoS Pathogens during this unprecedented global health crisis and extend our best wishes for their good health.

Major changes to the manuscript are summarized below:

(1) Additional quantitative analyses of immunoblots have been performed for Fig. 1 and Fig. 3 as per reviewer’s request. The data are presented in supplemental Figure S1-S5.

(2) Two panels have been added to Fig. 2 to demonstrate that Tax speckle structures (TSS), RNF8, and K63-linked polyubiquitin chains co-localize in both HeLa and U2OS cells. As previously reported (and ponited out in our response to reviewer 2’s comments), TSS also contains MDC1, BRCA1, DNA-PK, etc. The new results lend further support to the conclusion that TSS formation requires RNF8 and TSS represent misloclaized pseudo signaling scaffolds that sequesters repair facrtors and inhibits DNA damage response.
(3) A new panel (revised Fig. 3B) has been added to show that in pull-down experiment RNF8 interacts with WT (0.19) and M47 (0.14), but not with M22 (0.02) Tax. The extent of each form of Tax in interacting with RNF8 correlated directly with the ability to promote polyubiquitin chain assembly, NF-κB activation, TSS formation and DDR repression *in vivo*.

Specific comments of each reviewer are addressed point by point as below. Changes in the text are highlighted in yellow. We hope you find these modifications satisfactory and look forward to hearing from you in the near future.

With best regards,

Sincerely yours,

Chou-Zen Giam, Ph.D.

Professor of Microbiology and Immunology

**Reviewer #1:**

Overall, this an interesting study that contributes to our knowledge of the multiple roles of RNF8 in HTLV-1 infection. The following points will complement some of the data presented.

We thank the reviewer for the helpful comments and have addressed the points raised as follows.

1) In Figure 1A, quantification of several experiments would be helpful to visualize the DDR impairment induced by HTLV-1.

Quantitation of immunoblots in Fig. 1 has been done as suggested and added as supplemental information (Fig S1-S3). Additional blots performed for this experimental series include those in Fig. 3F and quantified (Fig. S5). The results are consistent across the board.

2) Why do the authors switch between HeLa-G delta-RNF8 and U2OS delta-RNF8 cells in Figure 2? Since the DNA damage experiments are shown in HeLa cells, it will make more sense to show colocalization of Tax and K63-pUb in the same cell lines.

U2OS cells are flat morphologically and produce excellent immunofluorescence images of DNA damage foci. As such, they are the cells of choice for DNA damage-related studies. To demonstrate the generality of the findings, we have added panels E and F to Fig. 2 to show that Tax and RNF8 co-localize in TSS in U2OS cells (2E), and TSS co-localize with K63-polyubiquitin chains in both HeLa (2F) and U2OS cells (2B).

3) The same question regarding Figure 2 also applies to Figure 3: will the TSS be similar in HeLa-G:deltaN-IKBalpha cells?

We expect this to be the case. ΔN-IκBα blocks NF-κB nuclear translocation at the end of the signaling cascade. It would neither disturb the assembly of cytosolic K63-polyubiquitin chains, nor affect the assembly of nuclear K63-polyubiquitin chains, which drive TSS formation.

4) The authors should determine whether Tax M47 and M22 interact with RNF8.

Tax has been shown to interact with RNF8 in S-tagged Tax pull-down experiment reported previously (<https://journals.plos.org/plospathogens/article?id=10.1371/journal.ppat.1005102>). To address the reviewer’s comment, we have performed a similar pull-down with S-tagged WT, M47, and M22 Tax. As expected, the strengths of interaction with RNF8 are in the rank order of WT ≥ M47 >> M22 (See revised Fig. 3B) and correlate directly with the stimulation of pUb chain assembly.

5) The new role of RNF8 in mediating activation of HTLV-1 transcription is interesting and could be further expanded. Will ectopic expression of RNF8 rescue the downregulation of LTR-Luc transcription observed in RNF8KD cells? The authors propose that TAK1 and downstream effects could be responsible for activation of the LTR promoter. Could they use a TAK1 inhibitor to support their claim? Tax levels could also be shown in the western blot in Figure 4D.

We agree with the reviewer about the importance of RNF8 activation in HTLV-1 viral transcription. However, we feel that identifying the exact signaling pathways and transcription factors involved requires an extensive analysis that is beyond the scope of the present paper.

**Reviewer #2:**

This study is interesting, but there are several concerns to be addressed.

We thank the reviewer for the helpful comments and have addressed the concerns as follows.

1) The authors show that RNF8 is required for appropriate DDR after genotoxic stress (Fig. 1B and C), and HTLV-1 infection impaired DDR even in the presence of RNF8 (Fig. 1A and D). They also think that Tax induced activation of RNF8, but DDR was impaired. These findings suggest that Tax suppresses the function of RNF8; however, there is little description about the mechanisms how activation of RNF8 by Tax impairs DDR. It is known that RNF8 interacts with MDC1, which is one of the components of Tax speckle structures (TSS). Does TSS contain RNF8 in HTLV-1-infected or Tax-overexpressing cells? If so, does Tax inhibit the interaction of RNF8 with other proteins in TSS, or its dissociation from the complex? It would be better if the authors could show some experimental data to suggest the mechanisms.

As shown in Fig. 2, TSS contain Tax, RNF8, K63-lined polyUb chains, and many other DDR factors. The reviewer is correct that RNA8 is recruited to ATM-phosphorylated MDC1 at the site of DNA damage. In unpublished data, we have found that both MDC1 and RNF8 associate with Tax. The presence of RNF8 in TSS is also stable and easily detected (see revised Fig. 2E & 2F). This contrasts with the weak and transient RNF8 signal in DNA damage foci (not shown). Therefore, we do not think Tax inhibits RNF8 interaction with other proteins. Rather Tax aberrantly activates RNF8 to assemble mislocalized pseudo signaling scaffolds that divert repair factors away from sites of DNA damage. The cytosolic signaling scaffolds, on the other hand, drive potent activation of not only IKK/NF-κB activation, but JNK, mTOR and other pathways that impact viral replication.

Although our proposed mechanism presented in the manuscript is that RNF8-mediated local ubiquitylation drives recruitment of DDR to TSS we are continuing to investigate finer details of this process. We demonstrated previously that in the absence of exogenously induced DNA damage Tax recruits a number of DDR factors including MDC1, BRCA1, DNA-PK to TSS (see <https://www.ncbi.nlm.nih.gov/pmc/articles/PMC2963403/figure/F1/>), which is spatially separable from ionizing radiation-induced speckles (IRIS)/DNA damage foci (see <https://www.ncbi.nlm.nih.gov/pmc/articles/PMC2963403/figure/F3/>) (see “The Viral Oncoprotein Tax Sequesters DNA Damage Response Factors by Tethering MDC1 to Chromatin. [S Mehdi Belgnaoui](https://pubmed.ncbi.nlm.nih.gov/?term=Belgnaoui+SM&cauthor_id=20729195) [^1^](https://pubmed.ncbi.nlm.nih.gov/20729195/#affiliation-1), [Kimberly A Fryrear](https://pubmed.ncbi.nlm.nih.gov/?term=Fryrear+KA&cauthor_id=20729195), [Julius O Nyalwidhe](https://pubmed.ncbi.nlm.nih.gov/?term=Nyalwidhe+JO&cauthor_id=20729195), [Xin Guo](https://pubmed.ncbi.nlm.nih.gov/?term=Guo+X&cauthor_id=20729195), [O John Semmes](https://pubmed.ncbi.nlm.nih.gov/?term=Semmes+OJ&cauthor_id=20729195), ***J Biol Chem***  2010 Oct 22;285(43):32897-905. <https://www.jbc.org/content/285/43/32897.long>). We have since mapped a region within MDC1 that binds Tax and note that this region overlaps reported RNF8 interacting sites. With these reagents we hope to provide in the near future additional insight into temporal and structural aspects of the sequestration and recruitment that drives formation of TSS.

2) In Fig. 3, the authors show that TaxM22 mutant could not induce TSS and impair DDR, indicating the NFkB pathway is involved in this machinery. The authors should discuss about the possible mechanisms for TSS formation by Tax-mediated NFkB activation.

The question on possible mechanisms of Tax action raised by the reviewer was addressed in the original manuscript and summarized schematically in Fig. 6. The mechanisms are further strengthened with new data to show that the RNF8 hijacked by Tax is responsible for the assembly of both nuclear K63-polyubiqutin and cytosolic K63-/M1-polyubiquitin chains. The former is responsible for TSS formation and the latter for NF-κB activation. M22 is unable to bind and activate RNF8 (see revised Fig. 3A&B), and as a result, defective in stimulating K63-lined pUb chain assembly, TSS formation (Fig. 3C&D), NF-κB activation (Fig. 3E) and DDR repression (Fig. 3F). This point is further discussed in the revised manuscript (page 13-14).

3) It is evident that RNF8 is down-regulated in ATLL cell lines and primary ATLL cells. The author reported that RNF8 is important for NFkB activation by Tax. Is there any correlation between expression level of RNF8 and activation of NFkB in those cells?

We have previously shown that RNF8 supports Tax-mediated NF-κB activation in a dose-dependent manner (<https://journals.plos.org/plospathogens/article?id=10.1371/journal.ppat.1005102>). The results have been mentioned, cited, and discussed in the revised manuscript (line 298-299).

**Reviewer #3**

The topic of the study is interesting and represents a logical extension of previous studies from the group. However some of the data do not convincingly support the conclusion of the authors and limit the scope of this work.

We think the reviewer may have missed the high-resolution images in the manuscript. We have highlighted those results by adding more explanations and references to alleviate the concerns.

1) Fig. 1: The statement “Notably, in the absence of bleomycin treatment, a low level of gH2AX was also observed in HeLa-G:DNIkBa:HTLV-1, but not in uninfected HeLa-G:DN-IkBa cells (compare Fig. 1A lanes 2 & 1)” (lines 132-133). This is not at all evident in the blot shown. Also the differences in panels B and C appear weak and unconvincing. Especially given these marginal differences, all immunoblots should be run in repeats, signals should be carefully quantitated and means and error bars should be shown. The number of repeats should be sufficient to support meaningful statistical analyses.

We refer the reviewer to the high-resolution images of both Fig. 1A ( γH2AX blot lane 2) and Fig. 1B (long exposure ( γH2AX blot lane). The link for the high-resolution image can be found at the right corner of each figure in the PDF.

All immunoblots have been run multiple times from independent experiments. Only representative figures are shown and quantified. Additional validation of the results can be seen in Fig. 3F.

2) Fig. 3 should also show RNF8 and γH2AX expression and localization.

We would like to emphasize that while TSS contain DNA repair factors, they are distinct from DNA damage foci. They are not structures that formed at sites of DNA damage and would **NOT** be expected to contain γH2AX. This point has been reported before (see “The Viral Oncoprotein Tax Sequesters DNA Damage Response Factors by Tethering MDC1 to Chromatin.” [S Mehdi Belgnaoui](https://pubmed.ncbi.nlm.nih.gov/?term=Belgnaoui+SM&cauthor_id=20729195) [^1^](https://pubmed.ncbi.nlm.nih.gov/20729195/#affiliation-1), [Kimberly A Fryrear](https://pubmed.ncbi.nlm.nih.gov/?term=Fryrear+KA&cauthor_id=20729195), [Julius O Nyalwidhe](https://pubmed.ncbi.nlm.nih.gov/?term=Nyalwidhe+JO&cauthor_id=20729195), [Xin Guo](https://pubmed.ncbi.nlm.nih.gov/?term=Guo+X&cauthor_id=20729195), [O John Semmes](https://pubmed.ncbi.nlm.nih.gov/?term=Semmes+OJ&cauthor_id=20729195), ***J Biol Chem***  2010 ;285(43):32897-905. <https://www.jbc.org/content/285/43/32897.long>). Based on the evidence provided in the manuscript, TSS are pseudo signaling structures assembled by nuclear RNF8 that is aberrantly activated by Tax, and they sequester repair factors into microscopically visible, but non-functional pseudo signaling complexes.

3) Most of the work presented is carried out in cell lines and only panel 5C shows expression data (from a published microarray dataset) in samples from ATL patients. It would be interesting to have data on the percentage of ATL cells in these PBMC samples and on the levels of expression of Tax and other viral genes in these samples.

 	As most ATL cells contain a single copy of proviral DNA per cell, the percentage of ATL cells in acute, chronic, and smoldering ATL can be estimated based on the proviral DNA loads (PVLs). A recent paper by Yonekura et al. (PMID:26134467) has reported the mean PVL per 100 PBMCs of acute, chronic, and smoldering ATL patients to be 85.7, 76.4, and 23.6. ATL cells therefore represent the majority (85.7% and 76.4% respectively) of the PMBC population in acute and chronic ATL patients. The Yonekura paper is cited in the legend to Fig. 5C of the revised manuscript.

It is well known that most ATL cells no longer express HTLV-1 viral genes, including Tax, *in vivo*.

**Minor Issues: Editorial and Data Presentation Modifications**

Reviewer #1:

1) Lane 177: "lanes 13" should be corrected.

We thank the reviewer for catching the error, which is now corrected.

2) Lane 214: pUbs assembly does not look reduced in Tax M47 compared to Tax wt (Fig. 3A). Quantification might help visualize the difference.

Quantitation has been done and included in the supplemental information. M47 is not as robust as WT in stimulating pUb chain assembly (see Fig. S4 for the quantitation of Fig. 3A)

3) Lane 220, Fig. 4D should be 3E.

We thank the reviewer for catching the error, which is now corrected.

Reviewer #2:

1) Fig.1 A-D: Differences and dynamics of the expression of gH2AX are not clear. Intensities of all bands should be quantified and normalized by an imaging software, and the dynamics of their expression levels should be shown as the graphs in the different panels of Figure 1.

Quantitation of the bands has been done and included as supplemental information.

2) Page 8, line 177: There is a typo in the terms “(lanes 1 & 2, also see Fig. 1A & B lanes 13 for comparison)”. There are no lanes “13” in Fig. 1A & B.

We thank the reviewer for catching the error, which is now corrected.

3) Page 10, line 220: “(Fig. 4D)” should be “(Fig. 3E)”. In addition, there is no explanation about Fig. 3D in the main text.

We thank the reviewer for catching the error, which is now corrected.

Reviewer #3: (No comments from this reviewer)
